# Bilateral calcification of the optic nerve sheath: A diagnostic dilemma

**DOI:** 10.1016/j.ajoc.2021.101106

**Published:** 2021-04-22

**Authors:** Yngvil Solheim Husum, Karoline Skogen, Petter Brandal, Pål Andre Rønning, Andreas Reidar Wigers, Johan Arild Evang, Øystein Kalsnes Jørstad

**Affiliations:** a Department of Ophthalmology, Oslo University Hospital, Norway; b Department of Radiology and Nuclear Medicine, Oslo University Hospital, Norway; c Department of Oncology, Oslo University Hospital, Norway; d Institute for Cancer Genetics and Informatics, Oslo University Hospital, Norway; e Department of Neurosurgery, Oslo University Hospital, Norway; f Section of Specialized Endocrinology, Oslo University Hospital, Norway; g Faculty of Medicine, University of Oslo, Norway

**Keywords:** Optic nerve sheath calcification, Optic nerve atrophy, Bilateral optic nerve sheath meningioma, Idiopathic duro-optic calcification

## Abstract

**Purpose:**

To present a case of symptomatic optic nerve sheath calcification and highlight clues and pitfalls for the final diagnosis: bilateral optic nerve sheath meningioma.

**Observations:**

A 48-year-old man presented with painless vision loss in his left eye and findings consistent with left optic nerve atrophy. Magnetic resonance imaging (MRI) displayed thinning of the left optic nerve without contrast-enhancement or evidence of compressive lesions. A supplementary computed tomography angiography (CTA) exposed scattered dural calcification, which included the optic nerves. This was regarded as an incidental finding. The initial diagnosis was ischemic optic neuropathy. Over the next two years, the vision loss in the left eye progressed. A CT of the orbits revealed extensive calcification surrounding both optic nerves. A second MRI was unchanged in comparison to the first MRI. The diagnosis was changed to idiopathic duro-optic calcification. The vision in the left eye further declined over another two years. Consecutive optical coherence tomography measurements of the peripapillary retinal nerve fiber layer suggested bilateral progressive thinning. A third MRI displayed progression of tubular contrast-enhancement surrounding the optic nerves. On the basis of this finding, the patient was finally diagnosed with a bilateral optic nerve sheath meningioma and received external beam radiotherapy.

**Conclusion and importance:**

It is crucial to differentiate an optic nerve sheath meningioma from idiopathic calcification of the optic nerve. In the present case the initial MRI did not detect optic nerve sheath abnormalities. To better demonstrate characteristic calcification, additional CT imaging should be considered when a bilateral optic nerve sheath meningioma is suspected.

## Highlights

• Harmless dural calcification can sporadically affect the optic nerve.
• Concurrent optic neuropathy should raise the suspicion of underlying pathology.
• Bilateral optic nerve sheath meningiomas commonly exhibit calcification.
• If a bilateral meningioma is suspected, CT better shows calcification than MRI.

## 1 Introduction

Calcification of the optic nerve sheath can be a harmless, incidental finding, but in the case of concurrent optic neuropathy, it may point to underlying disease. The term “idiopathic duro-optic calcification” has been used to describe cases of concurrent optic atrophy and optic nerve sheath calcification of unknown cause. We present a patient initially diagnosed with idiopathic duro-optic calcification. However, subsequent progression of optic neuropathy suggested an alternative diagnosis sharing similar clinical features to idiopathic duro-optic calcification, calling into question the existence of this entity.

## 2 Case report

**Fig. 1:**
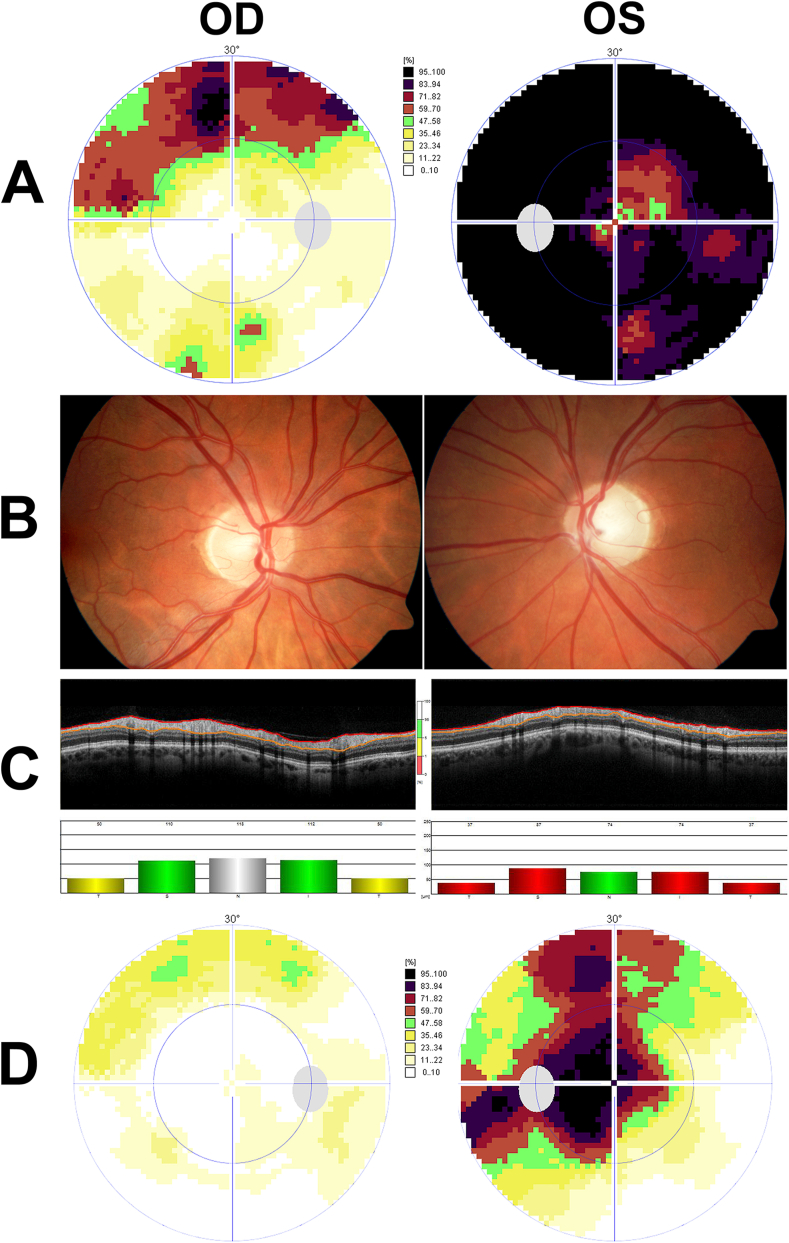
Threshold perimetry (A), fundus photography (B), and optical coherence tomography (OCT) (C) of a 48-year-old man presenting with painless loss of vision in his left eye. The intraocular pressure was 11 mm Hg in both eyes. Perimetry shows an upper arcuate defect in the right eye and deep visual field defects in the left eye. The right optic disc appears normal. The left optic disc is pale and displays mild cupping. On OCT of the peripapillary nerve fiber layer, the mean thickness is 97 μm (within the 95% reference range) in the right eye and 68 μm (below the 99% reference range) in the left eye. Threshold perimetry (**D**) after external beam radiotherapy to the optic nerves shows some improvement, with only mildly reduced sensitivity in the right eye and a remaining scotoma in the left eye.

A 48-year-old man presented to our neuro-ophthalmology section with painless vision loss in his left eye. Apart from tobacco smoking, his past medical history was unremarkable, and he was not taking any medication. Clinical examination revealed visual acuity of 20/20 in the right eye and 20/25 in the left eye. The patient could read 14 Ishihara test plates with the right eye but only seven test plates with the left. The left eye also exhibited a relative afferent pupillary defect. Confrontational visual fields were normal. Threshold perimetry was somewhat unreliable due to several false negative answers. It displayed deep visual field defects in the left eye. Perimetry also suggested an upper arcuate defect in the right eye, but this finding was not reproducible. The intraocular pressure was 11 mm Hg in both eyes. On fundoscopy, the right optic disc had normal color and a cup-to-disc (C/D) ratio of 0.2. The left optic disc was pale and had a C/D ratio of 0.4 (Fig. 1). The mean peripapillary nerve fiber layer (RNFL) thickness on optical coherence tomography (OCT) was 97 μm (within 95% reference range) in the right eye and 68 μm (below 99% reference range) in the left eye. Ultrasound of the optic nerve head did not suggest drusen. Fig. 1A–C displays findings at baseline.

The initial findings were consistent with left optic nerve atrophy, and neuro-imaging was requested. We performed magnetic resonance imaging (MRI) with a 1.5T system. The orbital sequences included 3D, T1-weigthed, fat-suppressed (FS) 1 mm scans with and without gadolinium contrast, axial T2-weighted 3 mm scans, and coronal T2-weighted, FS 3 mm scans. A cerebral time of flight (TOF) angiography was also performed.

The interpretation of the MRI was thinning of the left optic nerve without evidence of contrast-enhancement or compressive lesions. Additionally, the question was raised about an irregular lumen of the left carotid siphon, and we requested a supplementary cerebral computed tomography angiography (CTA) with iomeprol contrast. This examination showed only minimal atherosclerotic changes in both carotid siphons. There were also scattered dural calcifications encompassing the optic nerves, which were interpreted as an incidental finding. Subsequently, the patient underwent a full neurological evaluation without additional findings. He was discharged with a diagnosis of probable ischemic optic neuropathy, prescribed aspirin, and advised against further tobacco use.

**Fig. 2:**
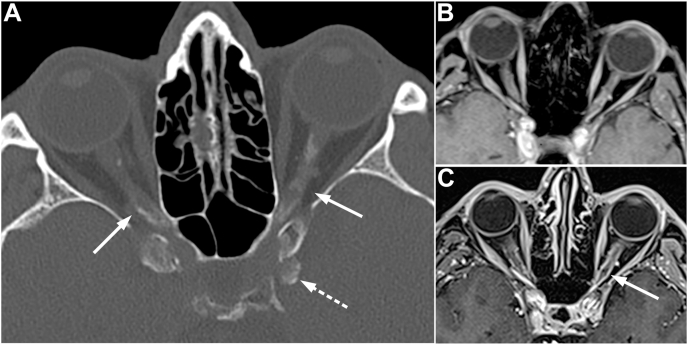
Neuroimaging of a 48-year-old man presenting with painless loss of vision in his left eye. A: Computer tomography shows calcification surrounding both optic nerves (arrows) and the left anterior clinoid process (dashed arrow). B: Initial magnetic resonance imaging (MRI) does not display contrast-enhancement or evidence of compressive lesions. C: Five years later, MRI displays progression of tram-track-like contrast enhancement surrounding the optic nerves (arrow). The final neuroradiological diagnosis was a bilateral optic nerve sheath meningioma.

A general ophthalmologist followed the patient. Two years later, however, he was re-referred to our neuro-ophthalmology section on suspicion of progressive optic neuropathy. The visual acuity was now 20/20 in the right eye and 20/32 in the left eye. Deterioration was not apparent on threshold perimetry or fundoscopy. OCT of the macular ganglion cell complex (mGCC) was carried out for the first time. The left eye displayed profound mGCC thinning with a mean thickness of 62 μm (below 99% reference range). Surprisingly, OCT also indicated mGCC thinning in the right eye with a mean thickness of 88 μm (borderline value). A second MRI was performed. The sequences were identical to the first examination, but an updated MRI system provided better images. The interpretation remained thinning of the left optic nerve without evidence of contrast-enhancement or compressive lesions. Eventually, the dural calcifications on CTA were questioned, and a dedicated orbital CT was requested. CT displayed extensive calcification surrounding the optic nerves and also calcification next to the left anterior clinoid process (Fig. 2). A detailed endocrine workup did not reveal phosphorus-calcium metabolism disorders. After reevaluation the diagnosis was changed to idiopathic duro-optic calcification.

The visual acuity remained 20/20 in the right eye but gradually deteriorated to 20/50 in the left eye over the next two years. Likewise, there was progressive dyschromatopsia in the left eye; the patient could still read all 14 Ishihara test plates with the right eye but none with the left. Worsening was not apparent on threshold perimetry or fundoscopy. However, the longitudinal change in mean peripapillary RNFL thickness was −2.3 μm/year in the right eye and −4.1 μm/year in the left eye. Declining visual function and thinning of the RNFL led to the question of whether progressive optic neuropathy was consistent with idiopathic duro-optic calcification.

A critical review of the literature about the entity disclosed a differential diagnosis with similar features: bilateral optic nerve sheath meningioma. Consequently, we requested a third orbital MRI. This examination displayed tubular contrast-enhancement surrounding the optic nerves (Fig. 2) as well as dural thickening adjacent to the left anterior clinoid process. In retrospect, subtle contrast-enhancement could also be seen on the second MRI. Taken together, the neuro-radiological findings over five years were: 1) progression of contrast enhancement surrounding the optic nerves with a tram-track appearance, 2) calcification of the optic nerve sheaths, and 3) development of dural thickening adjacent to the left anterior clinoid process. Based on these longitudinal findings, the final neuro-radiological diagnosis was a bilateral optic nerve sheath meningioma.

Because of progressive visual loss, the patient eventually received anti-neoplastic treatment in the form of external beam radiotherapy to the optic nerves (29 fractions of 1.8 Gy). Follow-up was delayed due to the COVID-19 epidemic, and the patient was first seen again nine months later. He then reported some visual improvement in the left eye following treatment. The visual acuity was still 20/20 in the right eye and had improved to 20/32 in the left eye. There was also some recovery on threshold perimetry, with only mildly reduced sensitivity in the right eye and a remaining central scotoma in the left eye (Fig. 1D). The patient will undergo further clinical and neuroradiological follow-up.

## 3 Discussion

We present the case of a patient with progressive visual loss and bilateral optic nerve sheath calcification. The patient was diagnosed with idiopathic duro-optic calcification, but subsequent progression disclosed a bilateral optic nerve sheath meningioma.

Soft tissue calcification is relatively common and can occur either as an age-related phenomenon or a disease manifestation. By convention, ectopic calcification diseases are classified based on the pathophysiological explanation; *metastatic* tissue calcification is caused by disorders of the phosphorus-calcium metabolism, whereas *dystrophic* calcification occurs in degenerated or necrotic tissue under normal phosphorus-calcium homeostasis. Finally, in *ectopic ossification* actual bone forms in other tissue types.^1^ With regard to the optic nerve sheath calcification in our patient, there was no evidence of metabolic disorder pointing to metastatic calcification. Dystrophic calcification of the optic nerves has been reported after trauma with hemorrhage, but a previous head injury could also be ruled out.^2^ Optic nerve sheath meningiomas can have multiple appearances on imaging; tubular expansion of the meninges surrounding the optic nerve is most common, but globular, fusiform, and focal optic nerve enlargement also exist.^3^ Nonetheless, the initial MRI did not reveal the underlying tumor, which temporarily led us to the false conclusion that the patient suffered from idiopathic duro-optic calcification.

Dural calcification is commonly an age-related, physiological finding without clinical relevance.^4^ Asymptomatic calcification can also affect the optic nerves and was first described in 1995*.*^5^ The following year, two symptomatic cases presenting with optic atrophy and bilateral optic nerve calcification were published.^6^ The authors proposed to group the cases under “calcific dural pathology” and introduced the term “idiopathic duro-optic calcification”. A few additional cases have since been reported.^7^, ^8^ However, Dr. Moseley questioned the original publication on idiopathic duro-optic calcification and pointed out that optic nerve calcification may be a presenting feature of bilateral optic nerve sheath meningiomas, which, unlike their unilateral counterparts, commonly exhibit calcification.^9^, ^10^ A critical review of the aforementioned case reports on idiopathic duro-optic calcification does not with certainty rule out the possibility of bilateral meningiomas; MRI was only performed in two cases (without information about contrast administration), not to mention the lack of histopathological verification in all cases. This raises the question of whether optic atrophy secondary to idiopathic duro-optic calcification represents an actual entity. Admittedly, the present case report also lacks histopathological verification, but radiological progression over five years supports the meningioma diagnosis, as does the modest clinical response to radiotherapy.

## 4 Conclusion

While harmless dural calcification can sporadically affect the optic nerve sheaths, concurrent optic neuropathy should raise the suspicion of underlying pathology. Despite modern imaging techniques, optic nerve sheath meningiomas can be notoriously difficult to diagnose.^11^ We could not detect either optic nerve sheath enhancement or calcification on the initial MRI. To better demonstrate characteristic calcification, additional CT imaging should be considered when a bilateral optic nerve sheath meningioma is suspected.^12^ A correct diagnosis has therapeutic implications, and it is imperative to differentiate an optic sheath meningioma from idiopathic calcification of the optic nerves. Idiopathic duro-optic calcification should only be used as a diagnosis of exclusion, if at all.

## Funding

The research was funded by 10.13039/501100003955Oslo University Hospital, Norway.

## Declaration of competing interest

The authors have no relevant conflicts of interest.

## References

[1] De Vilder E.Y., Vanakker O.M. (2015). From variome to phenome: pathogenesis, diagnosis and management of ectopic mineralization disorders. World J Clin Cases.

[2] Crompton J.L., O'Day J., Hassan A. (2004). Optic nerve calcification after trauma. J Neuro Ophthalmol.

[3] Parker R.T., Ovens C.A., Fraser C.L. (2018). Optic nerve sheath meningiomas: prevalence, impact, and management strategies. Eye Brain.

[4] McKinney A.M. (2017). Atlas of Normal Imaging Variations of the Brain, Skull, and Craniocervical Vasculature.

[5] Murray J.L., Hayman L.A., Tang R.A. (1995). Incidental asymptomatic orbital calcifications. J Neuro Ophthalmol.

[6] Phadke R.V., Agarwal P., Sharma K. (1996). Idiopathic duro-optic calcification - a new entity?. Clin Radiol.

[7] Utman S.A.K., Atkinson P.L., Smith R.A. (2011). Idiopathic dural optic nerve sheath calcification with intracranial parenchymal calcification. Eur J Radiol Extra.

[8] Ascaso F.J., Lasierra R. (2011). Idiopathic dural optic nerve sheath calcification associated with choroidal osteoma. Ophthalmic Surg Laser Imag.

[9] Moseley I. (1996). Idiopathic duro-optic calcification. Clin Radiol.

[10] Lewis T., Kingsley D., Moseley I. (1991). Do bilateral optic nerve sheath meningiomas exist?. Br J Neurosurg.

[11] Stunkel L., Newman N.J., Biousse V. (2019). Diagnostic error and neuro-ophthalmology. Curr Opin Neurol.

[12] Shapey J., Sabin H.I., Danesh-Meyer H.V. (2013). Diagnosis and management of optic nerve sheath meningiomas. J Clin Neurosci.

